# Sensitivity to Social Agency in Autistic Adults

**DOI:** 10.1007/s10803-020-04755-2

**Published:** 2020-11-17

**Authors:** Emma J. Morgan, Thomas Foulsham, Megan Freeth

**Affiliations:** 1 Psychology Department, University of Sheffield, Cathedral Court, 1 Vicar Lane, Sheffield, S1 2LT England; 2 Psychology Department, University of Essex, Wivenhoe Park, Colchester, CO4 3SQ England

**Keywords:** Autism, Social facilitation, Social agency

## Abstract

The presence of other people, whether real or implied, can have a profound impact on our behaviour. However, it is argued that autistic individuals show decreased interest in social phenomena, which leads to an absence of these effects. In this study, the agency of a cue was manipulated such that the cue was either described as representing a computer program or the eye movements of another participant. Both neurotypical and autistic participants demonstrated a social facilitation effect and were significantly more accurate on a prediction task when they believed the cue represented another participant. This demonstrates that whilst autistic adults may show difficulties in interpreting social behaviour this does not necessarily arise from a lack of sensitivity to social agency.

Humans are remarkably sensitive to the presence of other people. This sensitivity has been shown to lead to ‘social facilitation’ effects, whereby individuals show improvements in task performance when in the real or perceived presence of others (Dashiell 1935). However, autistic individuals have been shown to be less sensitive to behaviours derived from social partners, and typically show difficulties in identifying patterns of biological motion (Abell et al. 2000; Kaiser and Shiffrar 2009). Further, it has been argued that autistic individuals show decreased interest in social phenomena, which leads to an absence of social facilitation effects (Chevallier et al. 2012, 2014). However, exactly how this is manifested across a broad range of social cognition mechanisms is yet to be comprehensively explored. The aim of this study was therefore to investigate whether autistic adults are sensitive to the existence of others as independent social agents (hereafter termed ‘social agency’) and demonstrate social facilitation effects when they believe a cue to represent a social partner.

Neurotypical infants demonstrate a clear preference for social stimuli. When exposed to point light displays new-born babies can distinguish biological motion from non-biological motion, and preferentially attend to the biological motion display (Simion et al. 2008). Across development the attention we direct to the actions of our social partners becomes increasingly selective, with a narrowed focus directed to physical features capable of conveying social intentions e.g. pointing to an object with an index finger (Simion et al. 2008). This heightened sensitivity to the behaviour of others suggests that individuals may be significantly more attuned to social, as compared to non-social, entities (Loucks and Sommerville 2013). In line with these expectations the use of socially relevant stimuli has been shown to affect participants’ performance on a range of cognitive tasks including gaze cueing paradigms (Wiese et al. 2012), theory of mind tasks (Chevallier et al. 2014) and donation games (Izuma et al. 2011). Of note, neurotypical participants have been found to demonstrate ‘social facilitation effects’ in the form of improvements in performance brought about by the mere presence (real or implied) of another person (Chevallier et al. 2012, 2014; Hamilton and Lind 2016).

Recent research has shown that even simply manipulating the *perception* of a stimulus as being an independent social agent is sufficient to drive changes in behaviour, regardless of the physical appearance of the stimulus. Wiese et al. (2012) found that participants demonstrated significantly larger gaze cueing effects when they believed a face was controlled by a human agent as compared to a robot- regardless of whether the face they viewed was actually human. Further, in a key study, Gobel et al. (2018) aimed to investigate whether it is the physical appearance or the social relevance of a cue that elicits changes in participant behaviour. To do this they manipulated the perceived social agency of a cue, with the cue itself lacking any inherent social features. Participants were presented with a cue (a small red dot), which they were informed either indicated where another participant had preferentially looked during the same trial, or which had been selected at random by a computer program. It is important to note that the properties of the stimulus did not deviate between conditions, only the description of its nature was altered. The social manipulation was found to modulate inter-personal spatial orienting, with participants’ eye gaze aligning with the red dot significantly more when they believed the cue to have social agency. From this they concluded that attentional orienting in relation to social stimuli is not exclusively reliant on the physical properties of the stimulus but is strongly influenced by the perceived social agency of a stimulus. Taken together the studies conducted by Wiese et al. (2012) and Gobel et al. (2018) demonstrate that humans are sensitive to the social properties of a stimulus even in the absence of distinguishing physical characteristics, and that the use of social stimuli can lead to measurable changes in task performance.

However, whilst sensitivity to social agency is clearly a critical influence on behaviour in neurotypical populations, research would suggest that autistic individuals are less sensitive to identifying behaviour arising from other people than their neurotypical peers. For example, previous research has demonstrated that autistic individuals experience difficulties in discriminating patterns of biological motion (Klin et al. 2009; Kaiser and Shiffrar 2009). One explanation for this finding can be drawn from the ‘Bayesian’ and ‘Predictive Coding Framework’ accounts of perception. These accounts argue that individuals’ perceptual abilities are informed by ‘priors’ (prior contextual information) that allow us to generate specific predictions that guide our processing of perceptual stimuli (Pellicano and Burr 2012). An autism diagnosis is argued to lead to ‘hypo-priors’ where an individual is unable to successfully integrate their prior perceptual experiences, leading to an attenuation in their ability to make successful higher-order predictions, such as those necessary to identify patterns of biological movement (Van Boxtel and Lu 2013). Critically, such Bayesian models predict the behavioural changes that result when a stimulus is framed in a social vs non-social manner. The belief that one is interacting with a social partner is argued to generate a series of social priors that inform predictions relating to the partner’s next actions and preferences, which contribute to a social facilitation effect (Devaine et al. 2014). Therefore, if autistic individuals demonstrate difficulties with the integration of priors this may not only affect the identification of biological motion, but also lead to reduced social facilitation effects.

Of key importance, autistic individuals are not only believed to show difficulties in identifying social behaviour, they are also argued to be less sensitive to the *presence* of other people. Indeed, much previous research has indicated that individuals with an autism diagnosis do not demonstrate a social facilitation effect (Scheeren et al. 2010; Chevallier et al. 2012; Hamilton and Lind 2016). The ‘social motivation hypothesis’ of autism purports that autistic individuals have less interest in social phenomena than neurotypical individuals and therefore their behaviour is less likely to be affected by the social agency of a stimulus (Chevallier et al. 2012). For example, Chevallier et al. (2014) found that neurotypical children’s performance on a theory of mind task improved significantly when the task was administered by an experimenter rather than a computer, whereas autistic children did not show the same social facilitation effect. However, in contrast, a study conducted with autistic adults found that whilst they *did not* display social facilitation effects on a reputation management task, they *did* display social facilitation effects on a perceptual task when in the presence of an observer (Izuma et al. 2011). This suggests that autistic adults are not always inattentive to other people (as proposed by the social motivation hypothesis) and that it might depend on the particular task and context. The objective of the current research study is therefore to investigate the effect of manipulating the social agency of a cue in neurotypical and autistic adults. In particular, we investigated whether autistic adults show social facilitation when exactly the same abstract stimulus is interpreted as a social cue.

The current study took the form of an online experiment and was an adaptation of a paradigm previously used by Foulsham and Lock (2015). In one part of that study, participants completed a preference task in which they chose which of four abstract patterns they preferred, while their eye movements were recorded. In a subsequent, “guess” task participants were then asked to watch the eye movements of another participant (represented by animations of a moving red dot) and guess which image the other participant had chosen. The findings indicated that neurotypical participants were sensitive to the patterns of gaze behaviour and were able to accurately identify which design had been chosen by the previous participant. Without any explicit training, the guessing participants picked up on regularities in eye movements—such as the fact that people looked longer at the preferred item. In the present studies, we also investigated how well naïve neurotypical and autistic participants could predict preference based on the movements of a red dot—using the participant eye movement animations from Foulsham and Lock. However, unlike Foulsham and Lock’s study, the current studies contrasted ‘non-social’ instructions and ‘social’ instructions. During the ‘non-social’ part of the study, participants were informed that they would view an animation of a red dot, which represented a computer program selector, and that they would be asked to identify which of four designs they believed was selected by the program. In the ‘social’ part of the experiment the participants were instead informed that the red dot represented the eye movements of *another participant*, and they were then asked to choose which design that participant had selected.

In summary, this study aimed to investigate whether manipulating participants’ top-down perception of a cue as having social agency would affect performance on a prediction task. Based upon previous research (Wiese et al. 2012; Gobel et al. 2018) we predicted that neurotypical adults would be significantly more accurate at identifying which design was selected in the social, compared to the non-social condition. Since the visual information is the same in each case, this would be strong evidence of social facilitation. Of central focus, we predicted that autistic adults would not show the same improvement in accuracy as the neurotypical (NT) controls when party to the knowledge that the stimulus had social agency. Further to this, we predicted that autistic adults would find the social condition significantly more difficult to complete than NT adults, with previous research demonstrating that the social difficulties associated with autism are more pronounced when increasing the social complexity of a stimulus (Klin et al. 2002; Hanley et al. 2012). If autistic adults do not show the same improvement as NT controls in the social condition then this would support previous research demonstrating that autistic individuals are less sensitive to the social presence of others (Chevallier et al. 2012, 2014). However, if autistic adults do show improved performance given the knowledge that the cue presented has social agency, this would demonstrate that they can show social facilitation and therefore be sensitive to social agency.

## Method: Main Experiment

### Participants

The main experiment recruited 32 autistic participants (11 female, 21 male) and 32 age and gender matched Neurotypical (NT) Controls (11 female, 21 male). Autistic participants were recruited from the Sheffield Autism Research Lab (ShARL) database and received a gift voucher as a thank you for taking part. All Autistic participants had previously received a diagnosis of either ‘Aspergers’ or ‘Autism Spectrum Condition’ from a qualified clinician based in the UK. The NT participants were recruited via the online crowd-sourcing platform “Prolific” and received a monetary compensation as a thank you for their time. The study was approved by the Department of Psychology Ethics Committee, and all participants gave informed consent before participating. Three participants from the autistic group were excluded for failure to follow task instructions. A further two participants from the NT group scored more than 3 SD from the mean for task accuracy, indicating non-engagement with the task instructions, and were also removed from the analysis. All participants also completed the Social Responsiveness Scale (2nd Ed.) (SRS-2) as a measure of the level of social impairment associated with an autism diagnosis. Only NT participants that scored below the cut-off were included in the final sample. Three NT participants scored over the threshold for clinical relevance on the SRS-2 and so were excluded from the final analysis. Three autistic participants scored below the SRS-2 cut-off, however these participants had previously received a clinical diagnosis of autism from a registered clinician. Clinical judgement is a more reliable measure of diagnosis than self-report measures such as the SRS-2, and so these participants remained as part of the final sample. This left a total of 29 autistic participants, and 27 neurotypical controls (Table 1). An independent samples *t* test indicated a highly significant difference between groups on SRS-2 t-scores*, t*(37.5) = 9.643*, p* < .001, *d* = 3.15.^1^

**Table 1:** Participant characteristics

	Autistic participants	Neurotypical participants
Gender (male:: female)	19:10	19:8
Age		
Mean	35.9	36.7
SD	12.4	11.2
Range	21–60	23–58
SRS-2		
Mean	74.3**	52**
SD	11.5	4.7
Range	52–95	44–59

^**^Denotes significant between group difference, *p <* .001

### Design

The main experiment used a mixed-measures design with one between-subjects factor of ‘group’ (autistic or NT), and one within-subjects factor of ‘condition’ (non-social or social). All participants first completed the non-social part of the study, followed by the social part of the study. The order of conditions was not counterbalanced between participants in the main experiment as previous research has demonstrated that once a stimulus is identified as having social agency (e.g. representing eyes) participants are unable to disregard the attribution and therefore continue to show gaze cueing effects even if the cue is subsequently labelled as being non-social (Ristic and Kingstone 2005). However, in order to confirm that any differences observed between conditions in the main experiment could not be attributed to order effect two checks were performed: 1. Performance on the first half of the trials in each condition was compared to the second half of trials in order to establish whether practice effects via improvement over time were observed. 2. A control experiment was conducted which used a between-participants design with neurotypical participants whereby one group were informed that the red dot represented a computer program and a second, separate group were informed that the red dot represented another participant’s eye movements. The study paradigm was a prediction task, which required participants to predict which of four designs had been selected by an animated red dot.

### Materials and Apparatus

The study was conducted online through the use of the online survey platform ‘Qualtrics’. The stimuli in each trial were animations based on data collected by Foulsham and Lock (2015). In each case, four designs from freely available collections were displayed in a grid on a white background. These designs were abstract, colourful computer-generated artwork with no inherent meaning, ensuring that preferences for the designs were idiosyncratic. One hundred and forty-four designs were used in total and each design was randomly assigned to a group of four. Animations consisted of a red dot moving around the screen displaying the four designs. The trajectory of the red dot matched the eye movements of two representative participants (neurotypicals) from Foulsham and Lock (2015, Experiment 1) who were asked to view the four designs and indicate which of the designs they preferred. On average the duration of each clip was 4.3 s, and the clips were encoded as FLV files with a frame rate of 24fps. To ensure that the stimuli in each condition were equally difficult to predict, the two sets of source participant animations were counterbalanced between participants; each participant saw 18 animations deriving from one source participant in Part 1 (non-social), and 18 animations deriving from the other source participant in Part 2 (social). Thus, across the whole experiment, each particular animation appeared equally often in both conditions. Participants saw each animation only once and across the whole study, all participants viewed the same stimuli. The order of the animations was randomised in each part of the experiment.

Each participant also completed the Social Responsiveness Scale (2nd Ed.) (SRS-2), a 65-item questionnaire designed to test for the presence of social features associated with autism spectrum conditions. A clinical cut-off of a total t-score of ≥ 60 was used to distinguish between scores associated with neurotypical performance, and those associated with a diagnosis of autism (Constantino and Gruber 2012). The SRS-2 has demonstrated a high sensitivity (> 78%) to detecting social features associated with autism, and therefore was suitable for use in this study to confirm the range of social features present within the autistic group, and to control for individuals with high levels of social difficulties in the NT group.

### Procedure

**Fig. 1:**
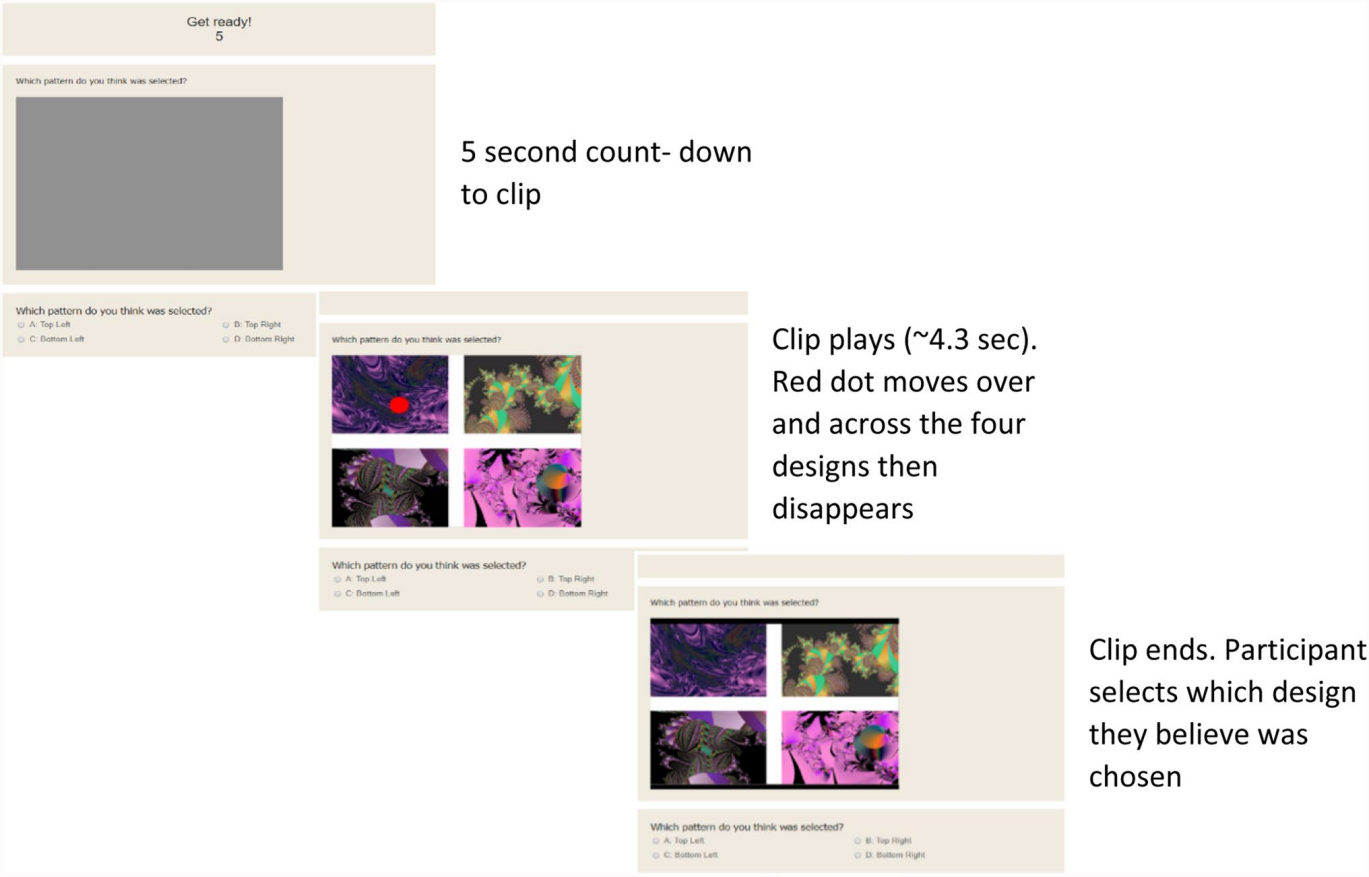
The trial procedure. Each trial displayed one animation and the order of trials was randomized

Prior to starting the main experiment each participant completed three practice trials. Each trial consisted of a 5 s countdown to a short clip, featuring the red dot moving over the four designs, after which it disappeared. Following the clip participants were presented with a list of each of the four designs and asked to select the design they believed had been selected by the red dot (Fig. 1).

During the practice trials the participant viewed each clip three times before being prompted to make a selection, but during the main part of the experiment the participant could only view each clip once prior to making the selection. The repetition of video clips during the practice trials allowed participants time to familiarise themselves with the presentation of the video stimuli prior to beginning the main experiment. The participant response was untimed, with each participant able to take as long as necessary to respond. For Part 1 participants were informed that “the red dot represents a computer program while it was selecting an image”; the non-social part of the study. Before starting each part of the experiment, the participant was asked to state what the red dot represented. Following completion of the non-social trials each participant was then asked to respond to a series of questions asking how easily they were able to guess which of the patterns was selected, and what was important in helping them make their decision (i.e. what strategies they used).

During the second part of the study participants were instead informed that “the red dot represents the eye movements of another participant while they were selecting an image”; the social part of the study. The participant was again asked to confirm what the red dot represented prior to starting the next part of the study. Participants did not receive feedback as to the accuracy of their selection in either the practice or main trials. Upon completion of the second part of the study the participants were again asked to confirm the ease with which they could identify the chosen design, what strategies they used to aid the identification and, additionally, if they had noticed any differences between the two parts or used different strategies in each part of the study. As the recruitment documents did not include any reference to the use of eye movements as part of the experimental stimuli (participants were simply informed that the study aimed to investigate how people used information from a cue to help them complete a task) participants were then fully debriefed as to the aims of the study.

## Results: Main Experiment

### Group Accuracy in the Social and Non-Social Condition

**Fig. 2:**
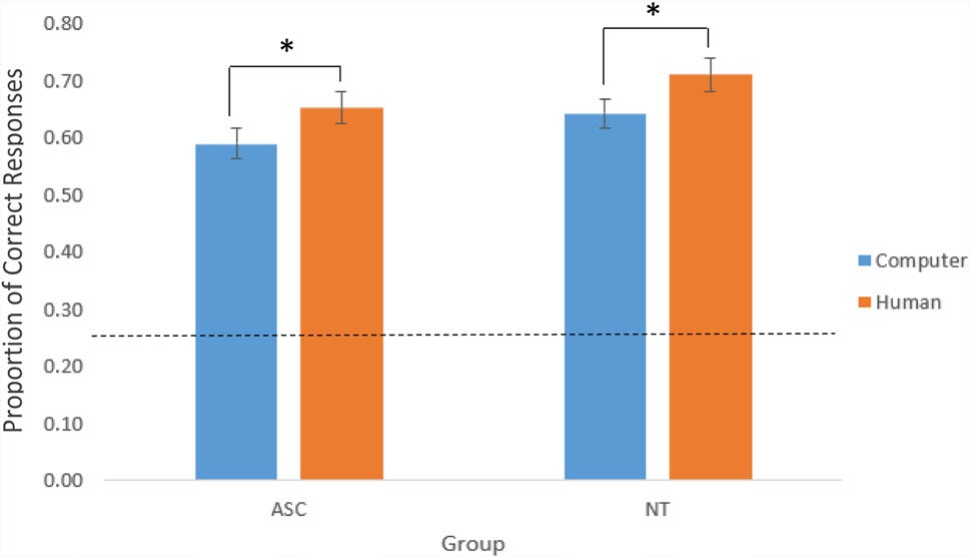
The proportion of correct responses for each group in each condition (social/non-social, ASC/NT). Error bars show +/−1 within-subject standard error of the mean (S.E.M). The dashed line indicates chance performance in this 4-alternative choice task

Accuracy was determined by comparing the participant’s guess with the choice made by the original, eye-tracked participant. The proportion of correctly identified designs was determined for each participant by calculating how many designs they correctly identified out of the total number of trials in each condition. Shapiro-Wilk tests of normality revealed that the data was not normally distributed (*p*s *< .*05). However, previous research has indicated that ANOVAs are consistently robust to violations of normality both within and across groups (Blanca et al. 2017). Therefore, an ANOVA was judged to be the most appropriate test for this analysis. A 2 × 2 mixed model ANOVA, with a within-subject factor of condition (social/non-social) and a between-subjects factor of group (autistic/NT) on the proportion of correctly chosen designs revealed a main effect of condition (*F*(1,54) = 10.096, *p =.*002, *ηρ*^*2*^ =.158), as the proportion of correct responses was greater in the social condition (M = .68, SD = .13) compared to the non-social condition (M = .61, SD = .12 ). There was also a main effect of group (*F*(1,54) = 4.896, *p = .*031, *ηρ*^*2*^ = .083), as the proportion of correct responses was greater for the NT group (M = .68, SD = .05) compared to the autistic group (M = .62, SD = .12). The accuracy across all conditions (approximately 60–70%) is similar to that observed previously in NT participants by Foulsham and Lock (2015) and indicates that participants can efficiently interpret the abstract moving dot in most trials. There was no condition x group interaction (*F*(1,54) = .014, *p = .*906, *ηρ*^*2*^ < .001), demonstrating that there were no significant differences between the groups in how they responded to the manipulation of the perception of the stimulus as social or non-social. Paired samples *t* tests confirmed that both the autistic (*t*(28) = − 2.265, *p =* .031, *d* = .391) and NT (*t*(26) = -2.226, *p* = .035, *d =* .684) group were significantly more accurate at predicting which design was chosen in the social, compared to the non-social, condition (Fig. 2).

### Ease of Completion

**Fig. 3:**
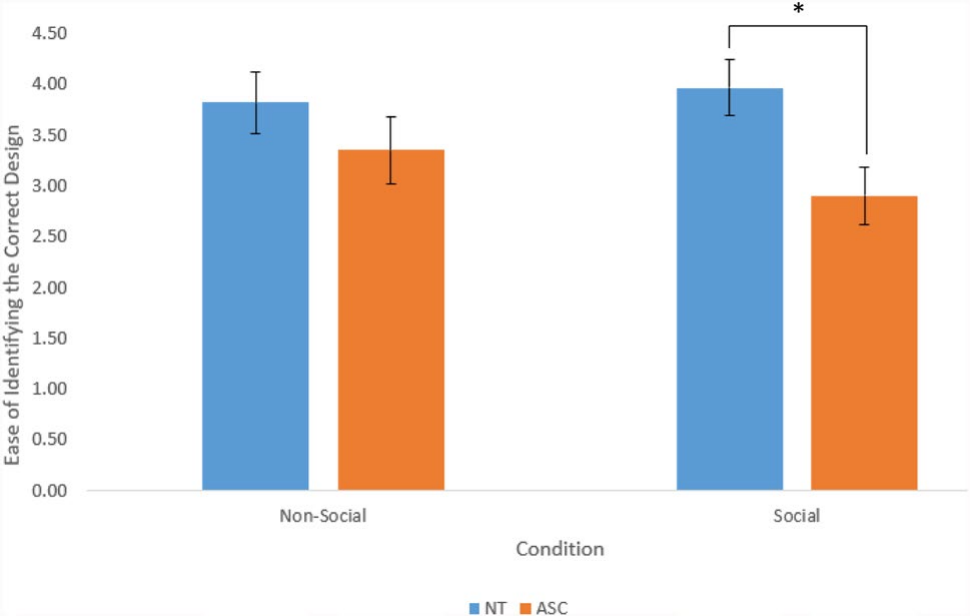
The ease of identifying the correct design in each condition for each group (non-social/social). Error bars show +/−1 within-subject standard error of the mean (S.E.M)

At the end of each part of the study participants were asked to rate on a Likert scale ‘How easy did you find it to guess which of the patterns was selected?’ The scale ranged from 1—‘Very Difficult’ to 7—‘Very Easy’. Planned comparisons investigated whether the NT and autistic groups differed in the ease with which they reported completing the task in the social or non-social conditions. Shapiro-Wilk tests of normality revealed that the data was not normally distributed (*p < .*05); therefore Mann-Whitney U tests were used. The results revealed that in the social condition autistic participants (Med = 2.00) reported finding it significantly more difficult to identify the chosen design then the NT participants (Med = 4.00; U =− 2.604, *p = .*009, *d* = .73). In contrast, in the non-social condition participants did not display this effect and there was no difference in reported difficulty between the autistic (Med = 3.00) and NT groups (Med = 3.00; U = − 1.263, *p = .*207, *d* = .34)*.* Therefore, despite the fact that the only change made to the stimuli was the way in which they were described (eye movements vs computer program), the groups differed in their estimates of difficulty (Fig. 3). The NT participants reported finding the second block easier, whereas autistic participants reported finding the social condition more difficult (when, in fact, they were also better at accurately predicting which design had been selected in the eye-movement condition than in the computer program condition).

In order to test whether individual perceptions of difficulty were related to how well each person performed on the task, Spearman’s Rho correlations were conducted between the self-rating of task difficulty and actual task performance. The results revealed that ease of completion did not correlate with task accuracy in the social condition for either the NT (r = .024, *p = .*906) or autistic (r = .154, *p = .*425) group. Likewise, there was also no correlation between ease of completion and task accuracy in the non-social condition for either the NT (r = − .146, *p = .*467) or autistic (r = .194, *p = .*314) group. This suggests that individual differences in perceived task difficulty did not reflect the NT or autistic groups’ accuracy in either the social or non-social condition, further demonstrating that the lack of confidence of the autistic participants in their own performance on the social task was not warranted.

### Social Responsiveness Scale Scores

To investigate whether task accuracy was related to the level of self-reported social difficulties, Pearson’s correlations were used to assess the relationship between task performance and SRS-2 t-scores. In order to assess the difference in performance between the first and second part of the study, a difference score was calculated by subtracting the accuracy scores for the non-social condition from the accuracy score for the social condition. SRS-2 t-scores were not significantly correlated with the difference score for either the neurotypical participants (r = .318, *p =* .106) or the autistic participants (r = .054, *p =* .782). Therefore, sensitivity to the social agency of the cue was not related to the level of social impairment shown by either autistic or neurotypical participants.

### Strategy Use in the Social Agency Attribution and Non-Social Agency Attribution Condition

We aimed to test whether the autistic and NT groups used different strategies in order to identify the chosen design. Upon completion of each condition participants were asked to identify the strategies they used in order to select their chosen image. Participants were presented with a pre-determined list of potential strategies and asked to identify as many strategies as applied. The proportion of respondents who reported using each strategy in each condition is shown below (Table 2).

**Table 2:** Percentage of participants in the autistic and NT groups who used each strategy to identify the chosen design in the social and non-social condition. Text in brackets indicates alternate phrasing used in the social part of the study.

	Control		Autistic	
Part 1 Question (Part 2 Question)	Non-social (%)	Social (%)	Non-Social (%)	social (%)
What I thought was the best item	4	4	3	3
Where the dot moved (Where the person looked)	44	41	62	52
How long or how much the dot selected a pattern (they looked)	26	37	31	48
Where the dot moved first (What they looked at first)	4	7	0	3
Where the dot moved last (What they looked at last)	89	85	79	62
Where the dot didn't move (What they didn't look at)	11	15	7	7
My previous knowledge about computers (about people)	7	0	14	3
I guessed	7	7	10	10
Other	0	4	0	0

Visual inspection of the strategy-use percentages indicated that each group used similar strategies, with the three most commonly used strategies in both the autistic and NT group being ‘where the dot moved/where the person looked’; ‘how long or how much the dot selected a pattern/they looked’; and ‘where the dot moved last/where they looked last’. This indicates that both participant groups interpreted the movements of the stimulus cue using comparable strategies, and that these strategies remained comparable across the groups in both the social and non-social conditions.

### Order Effects

Next, we aimed to determine that the improvement between the first and second part of the study was not explained by order effects arising from participants having already completed the same task in the non-social condition prior to the social condition. We therefore compared performance between the first half of the trials and the second half of the trials for each part of the study. A Shapiro-Wilk test of normality confirmed that the data was not normally distributed (*p < .*05). Wilcoxon Signed Rank tests revealed that there was there was no significant difference in accuracy between the first and second half of the trials in either the non-social or social conditions for the autistic and neurotypical groups respectively (*p* > .05). For both groups overall, there was no significant difference in accuracy in the non-social condition between the first half of the trials (Med = 0.67) and the second half of the trials (Med = 0.67; Z = -.02, *p =* .982, *d* = .01). There was also no significant difference in accuracy in the social condition between the first half of the trials (Med = 0.67) and the second half of the trials (Med = 0.67; Z = .28, *p =* .778, *d* = .08).

This analysis suggests that the significant improvement in accuracy between the non-social and social conditions is not explained by order effects. However, to confirm that the effect found in this main experiment was due to the manipulation of the cue, we conducted a control experiment which investigated whether participants who only believed a cue to represent the eye movements of another participant were significantly more accurate at the task than a separate group of participants who only believed the cue to represent a computer program.

## Method: Control Experiment – Order Effects Check

### Participants

Based on a power calculation conducted using the observed effect from the main experiment, the control experiment recruited 38 neurotypical participants for the non-social group (30 Female, M = 30.76, SD = 9.64, Range = 18-60), and recruited 36 age and gender matched participants for the social group (30 Female, M = 30.41, SD = 9.42, Range = 19-57). Participants were recruited via the online crowd-sourcing platform “Prolific” and received a monetary compensation as a thank you for their time.

Five participants were excluded who had either received a previous diagnosis of an autism spectrum condition, or who were awaiting an official diagnosis. A further 4 participants were excluded for failing to follow the task instructions (n = 3), or having prior knowledge of the task (n = 1). For the non-social group this left a final sample of 34 participants (28 Female, M = 30.77, SD = 9.96, Range = 18-60); for the social group this left a final sample of 31 participants (25 Female, M = 30.26, SD = 9.62, Range = 19-57).

### Design

The control experiment used a between-participants design, with one independent factor of ‘group’ (non-social or social). The control experiment used the same design and apparatus as outlined for the main experiment. This was an internal replication and was pre-registered on the Open Science Framework (osf.io/yr8n3).

### Procedure

The control experiment used a similar procedure to the main experiment. However, each participant completed *only* the non-social or social part of the experiment, completing 18 trials in total. As in the main experiment, upon completion of the 18 experimental trials the participants were asked to confirm the ease with which they could identify the chosen design.

## Results: Control Experiment—Order Effects Check

**Fig. 4:**
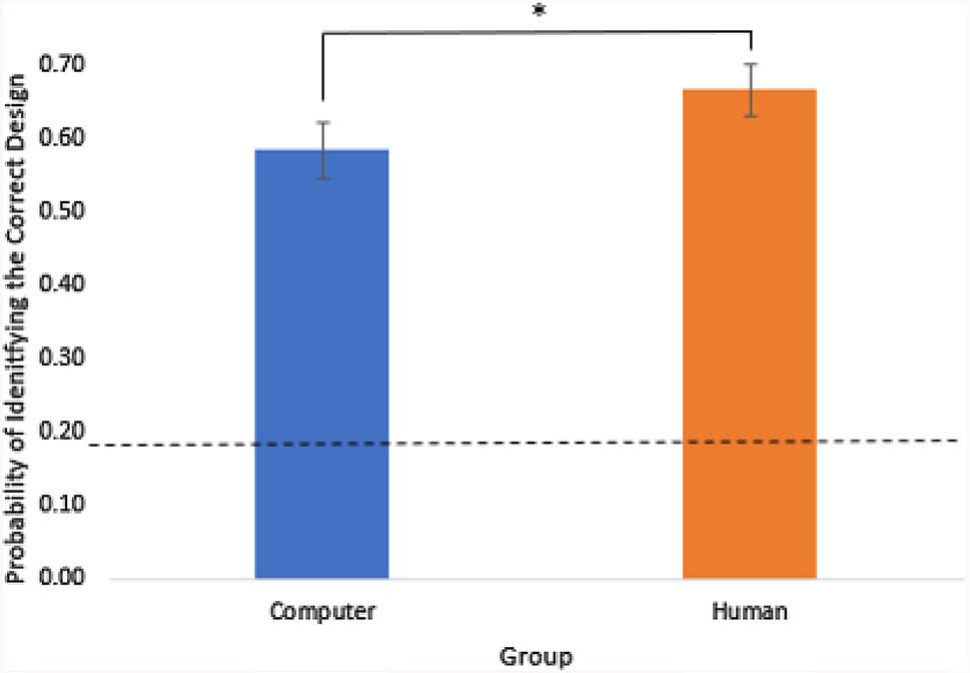
The proportion of correct responses for each group (non-social/social). Error bars show +/−1 within-subject standard error of the mean (S.E.M). The dashed line indicates chance

The aim of the control experiment was to check that the difference in accuracy between the conditions was due to the experimental manipulation and not an order effect. A Shapiro-Wilk test of normality confirmed that the data for the social group was not normally distributed (*p = .*001). Mann-Whitney U tests revealed a significant difference in accuracy between the non-social group (Med = .58) and social group (Med = .67; U = 2.26, *p = .*024, *d =* .58), with participants in the social, eye movement, group significantly more accurate at predicting which design was chosen (Fig. 4). This result is completely consistent with the main experiment, showing a robust, approximate 10% increase in accuracy, in two separate participant samples. This confirms that participants performed significantly more accurately when they believed the cue to possess social agency, and that the significant improvement in task accuracy between the non-social and social condition in the main experiment is not explained by order effects.

## Discussion

This study investigated whether manipulating participants’ perception of a cue as having social agency would affect autistic adults’ performance on a prediction task. As predicted, NT participants were significantly better at identifying the correct design when they believed that the cue represented the eye movements of another participant compared to when they believed the cue to be generated by a computer program and therefore not to have social agency. However, in contrast to the study hypotheses, the results clearly demonstrated that autistic participants also showed a significant improvement when they believed a cue to have social agency, even though they reported finding the social condition significantly more difficult to complete than did the NT participants. Further, prediction accuracy was not related to individual differences in social impairment, as indicated by the SRS-2. This therefore provides evidence that autistic adults show the same social facilitation effect as neurotypical adults and can more accurately predict another’s choices given the knowledge that a cue has social agency. This demonstrates that whilst autistic adults may show difficulties in interpreting patterns of social behaviour this does not necessarily arise from a lack of sensitivity to social agency.

The finding that NT participants were significantly more accurate at predicting which design would be chosen in the social condition when they believed that the dot represented eye movements supports a number of previous studies. Firstly, it supports those studies which demonstrate that a cue does not need to display visual social characteristics, such as eyes, or biological motion, in order for participants to show behavioural differences. Instead it appears that simply believing a cue to possess social agency is sufficient to generate social facilitation (Wiese et al. 2012; Gobel et al. 2018). Secondly, as this improvement does not rely upon the physical properties of the stimulus this study therefore lends support to research suggesting that these changes occur as a result of the engagement of theory-of-mind processes (Hamilton and Lind 2016) which allow inferences into the mental state of a social partner and promote increased accuracy on the prediction task.

This study shows that autistic adults were significantly more accurate at identifying which design would be chosen when they believed a cue to have social agency, demonstrating a similar sensitivity to the social properties of a stimulus as neurotypical individuals. Task performance was found to be unrelated to the level of social impairment associated with an autism diagnosis (as measured by the SRS-2). Of key interest, the finding that autistic adults did spontaneously take account of the social agency of the cue contrasts with the social motivation hypothesis of autism (Chevallier et al. 2012). The social motivation hypothesis proposes that a lack of engagement with social stimuli may actually be a causal factor of the social difficulties associated with autism, rather than a side effect. However, in contrast to this theory the findings from our study suggest that autistic adults do show comparable social facilitation effects to NT adults, clearly demonstrating that the perception of a cue as being social significantly improved prediction accuracy in both groups.

One potential explanation for improved performance when provided with the critical information that the cue possessed social agency could arise from the study’s use of a disembodied stimulus. The use of the red dot allowed for the control of extraneous variables arising from the physical characteristics of typical social cues. The social difficulties associated with autism are more pronounced when using increasingly complex social stimuli, for example, moving from the use of a photograph to a dynamic video (Klin et al. 2002), and autistic individuals display gaze avoidance behaviour, which is thought to affect their ability to process social cues (Hanley et al. 2014; Freeth and Bugembe 2019). The use of a disembodied stimulus may have served to remove the aversive physical appearance of the eyes; thereby allowing autistic participants to process the social information provided in the second part of the study without the distraction of the physical properties of the stimulus. This is important as it suggests that behaviours associated with autism can present differently as a consequence of the stimuli used within a given paradigm, this may therefore exaggerate the extent of a difficulty by masking preserved underlying abilities.

Whilst autistic participants’ task performance was improved by the perception of the cue as social, they were significantly less accurate than the NT participants in both the non-social and social condition. One explanation for this finding can be drawn from the Bayesian account and the predictive coding framework of perception (Pellicano and Burr 2012; van Boxtel and Lu 2013). These accounts argue that an overreliance on lower-order (local) processing due to a decrease in higher-order (global) processing leads to difficulties in identifying the ‘bigger picture’. In addition to the dynamic red dot cue, each trial also featured four background patterns displaying abstract visual details. To successfully complete each trial the participant therefore had to rely on global processing in order to integrate the specific details of the scene to form a ‘big picture’ that allowed the recognition of the pattern chosen by the red dot. If autistic individuals focus more on local, specific details, and experience difficulties in global processing then it is likely that this affected their ability to integrate all of the information available in the scene. This therefore would have made it harder to predict the preference of the cue in either the social or non-social condition, leading to the autistic participants being less accurate then the NT participants in both conditions.

A further finding arising from this study relates to the ease with which participants reported being able to complete each part of the experiment. There was a significant difference between groups for the ease with which they reported completing the social part of the study. Specifically, the autistic group reported finding the social condition significantly more difficult to complete than did the NT group. However, one question which arises from this finding is whether the autistic participants actually did find the social part of the task harder to complete than the NT participants, or whether they just perceived it to be so. Self-ratings of task difficulty did not correlate with task performance for either the autistic or NT group, suggesting that an individual’s perception of the difficulty of the task did not reflect their actual performance. An explanation for this finding could stem from the presence of demand characteristics (Orne 1962; Nichols and Maner 2008). There is a general awareness that autism is typically associated with difficulties in social cue use. This awareness may therefore have led to the autistic participants forming expectations regarding their own abilities, and thus to the generation of demand characteristics when rating the difficulty of the social task, whereas in reality knowledge that the cue had social agency actually improved performance. The evidence provided by the current study indicates that, in relation to this social agency task at least, this lack of self-confidence demonstrated by autistic participants is not warranted.

One limitation of this study is that the method of data collection did not allow for accurate recording of response time data. Such data would have allowed further insights into the differences between the autistic and neurotypical groups in how they rated the difficulty of the task in the social condition. Further, it is unlikely that this sample is representative of the autistic population as a whole. Autism spectrum condition is a heterogenous diagnosis and whilst the participants recruited for this study are clearly sensitive to social agency there is the potential that some autistic participants may not show a similar sensitivity to the presence of a social partner. Importantly, however, the findings of this study clearly indicate that the assumption of a lack of sensitivity to social stimuli is certainly not representative of the capabilities of all autistic individuals. In summary, the results of this study reveal that manipulating the perception of a disembodied stimulus as either ‘social’ or ‘non-social’ was sufficient to drive changes in both the NT and autistic participants’ behaviour, positively affecting their performance on the prediction task. In line with the current focus on increasing ecological validity in social cognition research, future research should therefore aim to expand upon the findings of this paper through the use of more complex social stimuli in order to determine if these findings extend to more ecologically valid contexts.

## Conclusion

This study investigated whether manipulating the perception of a cue as being socially relevant would affect autistic participants’ performance on a prediction task. The results clearly demonstrate that both autistic and neurotypical adults were significantly more accurate when they believed a cue to have social agency. Strikingly, this effect occurred despite the visual stimulus remaining exactly the same across both conditions. The results from this study therefore clearly illustrate that autistic adults can demonstrate a social facilitation effect and, therefore, that autistic adults in general are sensitive to social stimuli portraying other people’s actions when completing a prediction task.

## Data Availability

The data that support the findings of this study are available in the Open Science Framework at https://osf.io/yr8n3/?view_only=8fcd50894ede4d099b502312eefc0f1f

## Footnotes

1 Inclusion of the NT participants who scored above clinical relevance on the SRS-2 does not alter the nature of the outcome of any of the statistical analyses discussed in this paper.
